# Organic Connection of Holobiont Components and the Essential Roles of Core Microbes in the Holobiont Formation of Feral *Brassica napus*

**DOI:** 10.3389/fmicb.2022.920759

**Published:** 2022-07-08

**Authors:** Seong-Jun Chun, Yingshun Cui, Su-Hyang Yoo, Jung Ro Lee

**Affiliations:** ^1^LMO Team, National Institute of Ecology, Seocheon, South Korea; ^2^Cell Factory Research Center, Korea Research Institute of Bioscience and Biotechnology, Daejeon, South Korea

**Keywords:** *Brassica napus*, plant holobiont, microbial network, recurrent pattern, natural ecosystem

## Abstract

*Brassica napus* (Rapeseed) is an econfomically important oil-producing crop. The microbial interactions in the plant holobiont are fundamental to the understanding of plant growth and health. To investigate the microbial dynamics in the holobiont of feral *B. napus*, a total of 215 holobiont samples, comprised of bulk soil, primary root, lateral root, dead leaf, caulosphere, basal leaf, apical leaf, carposphere, and anthosphere, were collected from five different grassland sites in South Korea. The soil properties differed in different sampling sites, but prokaryotic communities were segregated according to plant holobiont components. The structures of the site-specific SparCC networks were similar across the regions. Recurrent patterns were found in the plant holobionts in the recurrent network. *Ralstonia* sp., *Massilia* sp., and *Rhizobium* clusters were observed consistently and were identified as core taxa in the phyllosphere, dead leaf microbiome, and rhizosphere, respectively. Arthropod-related microbes, such as *Wolbachia* sp., *Gilliamella* sp., and Corynebacteriales amplicon sequence variants, were found in the anthosphere. PICRUSt2 analysis revealed that microbes also possessed specific functions related to holobiont components, such as functions related to degradation pathways in the dead leaf microbiome. Structural equation modeling analysis showed the organic connections among holobiont components and the essential roles of the core microbes in the holobiont formations in natural ecosystem. Microbes coexisting in a specific plant showed relatively stable community structures, even though the regions and soil characteristics were different. Microbes in each plant component were organically connected to form their own plant holobiont. In addition, plant-related microbes, especially core microbes in each holobiont, showed recurrent interaction patterns that are essential to an understanding of the survival and coexistence of plant microbes in natural ecosystems.

## Introduction

The interactions between plants and microbes have a profound effect on the growth, productivity, and health of plants (Trivedi et al., 2020). Plant growth-promoting rhizobacteria in the rhizosphere have been the subject of attention because of their abilities to produce phytohormones and siderophores, solubilize phosphorus, fix nitrogen, and improve resistance to pathogens (Lugtenberg and Kamilova, 2009). Recently, many studies have been conducted into microbial communities, not only in the rhizosphere but also throughout plant organs, and the concept of a plant holobiont—the host and its symbiont—has emerged (Vandenkoornhuyse et al., 2015). Multiple microbes inhabit the inside and outside of the plant, as well as its roots, and the diversity and functions of these microbiomes are essential to understanding plant biology and the ecosystems in which the plants are involved. Plant holobiont dynamics are mainly influenced by the host characteristics, the surrounding microbiome, and the environment (Vandenkoornhuyse et al., 2015; Sánchez-Cañizares et al., 2017). However, there has been little research into the dynamics of the plant holobiont across the niches represented by multiple plant organ types (Cregger et al., 2018; Hassani et al., 2018).

Rapeseed (*B. napus* L.), is an important source of vegetable oils, animal feed, and biodiesel. Various cultivars have been developed to increase the quality and amount of oil produced, and to increase the resistance of the plant to herbicides or insect herbivores (Lombardo et al., 2020). Recently, many studies have focused on the microbial communities associated with *B. napus*. Rochefort et al. (2019) and Taye et al. (2019) found that core taxa were consistent across their sites and sampling periods, and that small genetic differences in *B. napus* can cause changes in the microbial content of the rhizosphere and seed microbiome. However, these studies involved samples from plants grown in controlled experimental fields. In South Korea, *B. napus* has mainly been grown as a representative landscape crop for spring festivals. Recently, however, the unintentional release of genetically modified rapeseed into natural ecosystems has been increasing, and the environmental threat posed by gene transfer during hybridization is also increasing (Kim et al., 2020). Therefore, information about the diversity of microbes coexisting and interacting with *B. napus* and their functions are essential to an understanding of the potential effects of transgenic rapeseed on the natural ecosystem.

In this study we investigated microbial community dynamics from bulk soil to flower within *B. napus* under different environmental conditions. We analyzed 215 different holobiont samples from five different grassland sites using amplicon 16S rRNA gene-targeted Illumina MiSeq sequencing. Recurrent microbial network analysis and PICRUSt2 analysis were performed to investigate the microbial interactions and functions in the holobiont of feral *B. napus.* Structural equation model was constructed to explore the interactions among holobiont components. The following questions were addressed in this study: “What are the differences and similarities of the holobiont components across the region?,” “Are there any consistent patterns and key players in the holobiont microbial community structure?,” “What are the ecological functions of each different holobiont?,” and finally “How are the holobiont components connected and what affects them?”

## Materials and Methods

### Study Site and Sampling Design

**Figure 1:**
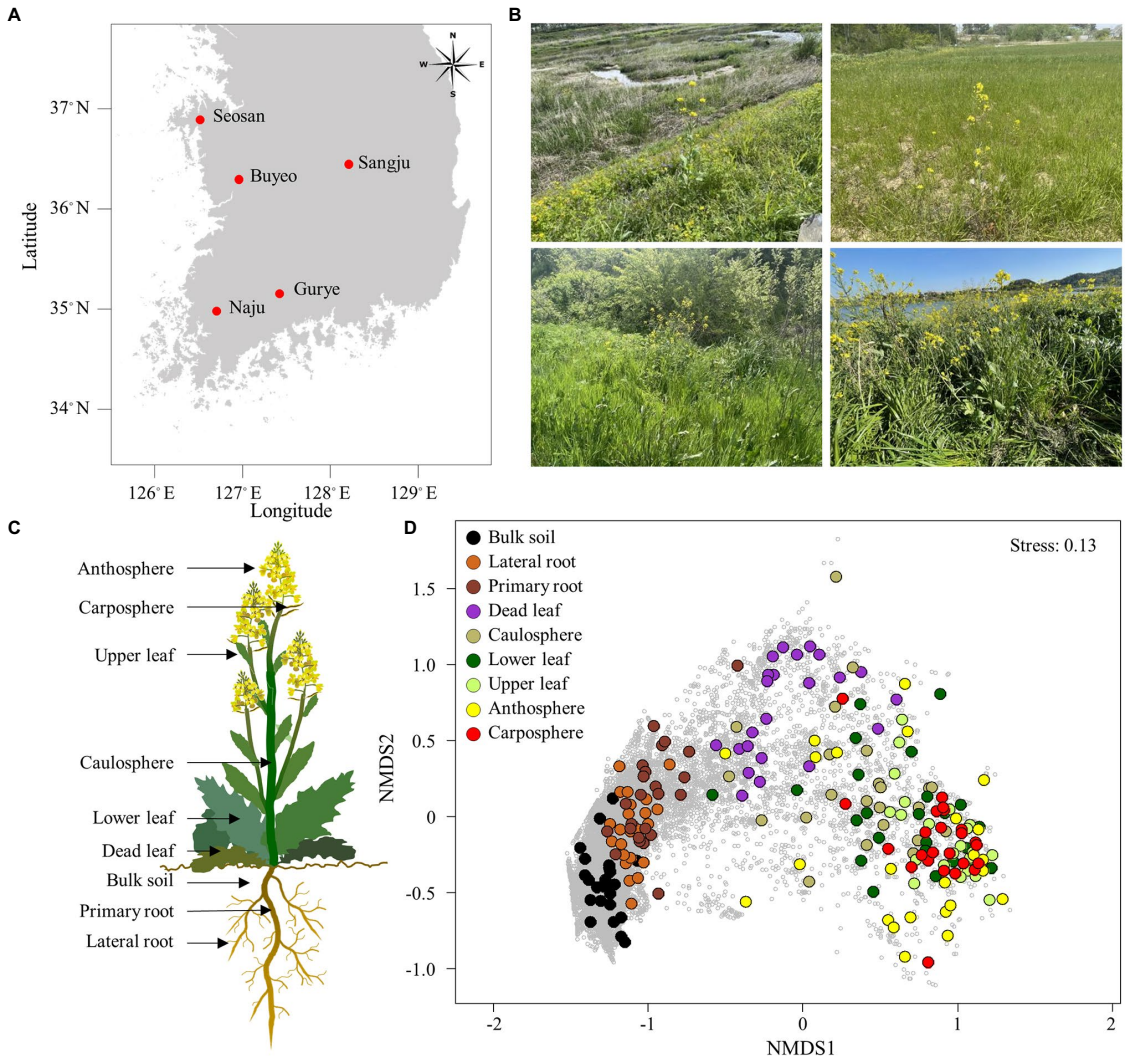
Sampling area and holobiont of *Brassica napus*. **(A)** Map showing the sampling sites. **(B)** Sampling sites with *B. napus*. **(C)** Holobiont samples collected in this study. **(D)** Non-metric multidimensional scaling (NMDS) ordination plot of Bray–Curtis community dissimilarities based on prokaryotic ASVs. Small gray circles represent ASVs.

Samples were collected between April 14 and April 30, 2021, from five different sites: Buyeo (36°9′12.21′N, 127°0′0.79′E), Gurye (35°13′41.69′N, 127°27′14.48′E), Naju (35°00′3.16′N, 126°42′7.58′E), Sangju (36°26′21.53′N, 128°15′32.85′E), and Seosan (36°42′35.04′N, 126°32′36.90′E; Figure 1A). Five different holobiont samples were collected in Gurye, Naju, Sangju, and Seosan, while four plant samples were collected in Buyeo. However, one dead leaf sample was not available in Buyeo. The minimum distance between sites was 70 km, and the maximum was 200 km. Sampling sites were selected to include natural habitats of *B. napus* with diverse plant species which had experienced low levels of disturbance by humans (Figure 1B). Plants at the flowering stage, of similar size, were selected, and after digging up each plant with an ethanol-sterilized shovel to minimize root damage, sampling was carried out for each holobiont component: bulk soil, primary root, lateral root, dead leaf, caulosphere, basal leaf, apical leaf, carposphere, and anthosphere (Figure 1C). Bulk soil samples were taken from the soil that fell off the plant following light shaking, and the parts that did not contain the plant debris and root were taken. After collecting the bulk soil samples, the plant was shaken vigorously to remove loosely bound soil, and the root part was divided into the primary root and lateral root. Roots and tightly bound soil were collected together. To collect healthy leaf microbiome samples, a 2 ml tube was used to punch out leaf disks at the various locations of the basal leaf (designated “lower leaf”) and the apical leaf (designated “upper leaf”). The leaves that turn from yellow to brown at the end of their life span were also collected as leaf disks (designated “dead leaf”). For caulosphere samples, stem sections were taken with a sterile blade 5–10 cm above the ground. Flowers and immature fruit were collected to investigate the anthosphere and carposphere. To minimize contamination, the shovel, forceps, and blades were cleaned with 70% ethanol and washed with sterile water between each sample. The samples were stored at −80°C until DNA extraction.

**Table 1:** Chemical and physical properties of soils in the sampling areas.

Sites	Gurye (*n* = 5)	Naju (*n* = 5)	Buyeo (*n* = 4)	Sangju (*n* = 5)	Seosan (*n* = 5)
Organic matter (%)	2.8 ± 1.0	3.0 ± 2.1	1.7 ± 0.3	1.3 ± 0.8	2.4 ± 1.0
Total nitrogen (%)	0.12 ± 0.04	0.12 ± 0.08	0.08 ± 0.02	0.06 ± 0.03	0.11 ± 0.05
AP (mg/kg)	147.8 ± 39.9	99.7 ± 38.9	126.8 ± 49.6	57.9 ± 17.8	73.6 ± 18.3
Ex. K (cmol/kg)	0.21 ± 0.06	0.31 ± 0.17	0.29 ± 0.02	0.13 ± 0.04	0.35 ± 0.24
Ex. Ca (cmol/kg)	4.7 ± 1.8	7.7 ± 2.4	7.7 ± 6.4	2.0 ± 0.2	5.8 ± 1.8
Ex. Mg (cmol/kg)	0.44 ± 0.22	0.92 ± 0.51	0.83 ± 0.1	0.36 ± 0.10	0.83 ± 0.29
Ex. Na (cmol/kg)	0.14 ± 0.02	0.21 ± 0.06	0.10 ± 0.02	0.14 ± 0.01	0.13 ± 0.02
CEC (cmol/kg)	7.0 ± 1.4	9.0 ± 4.5	7.8 ± 0.3	4.4 ± 2.0	8.1 ± 2.0
pH	6.6 ± 0.6	6.8 ± 0.7	7.0 ± 0.7	5.7 ± 0.2	6.6 ± 0.4
EC (dS/m)	0.076 ± 0.029	0.143 ± 0.072	0.093 ± 0.054	0.043 ± 0.012	0.099 ± 0.058
NaCl (%)	0.003 ± 0.001	0.006 ± 0.002	0.002 ± 0.000	0.003 ± 0.001	0.004 ± 0.001
Sand (%)	76.2 ± 6.9	60.4 ± 16.1	59.8 ± 1.3	80.8 ± 3.7	68.1 ± 11.6
Silt (%)	12.1 ± 3.8	22.7 ± 11.0	24.6 ± 2.7	9.4 ± 1.2	17.1 ± 8.4
Clay (%)	11.7 ± 3.2	16.8 ± 5.5	15.6 ± 2.1	9.8 ± 2.4	14.8 ± 3.3

Values are mean ± standard deviation. EX, exchangeable; CEC, cation-exchange capacity; AP, available phosphorus; EC, electrical conductivity.

Bulk soil samples were used for the analysis of soil characteristics. The soil organic matter, total nitrogen, available phosphorus, exchangeable cation composition (K, Ca, Mg, and Na), cation-exchange capacity, pH, electrical conductivity, NaCl concentration, and relative percentages of sand, silt, and clay were measured by the AT Analysis Center Co., Ltd. (Incheon, South Korea) in accordance with the National Institute of Agricultural Science and Technology (NIAST) soil and plant analysis methods (NIAST, 2000).

### DNA Extraction and Sequencing

DNA was extracted using DNeasy PowerMax^®^ soil kits (Qiagen, Hilden, Germany) according to the manufacturer’s instructions, and the quality and concentration of extracted DNA were checked using a NanoDrop 2000 spectrophotometer (Thermo Scientific, Wilmington, DE, United States). The prokaryotic 16S rRNA gene was amplified using a universal prokaryotic primer set with overhang adapter sequences, 342F/806R (342F: 5′-CTACGGGGGGCAGCAG-3′; 806R: 5′-GGACTACCGGGGTATCT-3′), which targets the V3-V4 region of the 16S rRNA gene (Mori et al., 2014). PCR amplification, purification, and quantification were performed according to the method described in our previous report (Chun et al., 2019). Briefly, TaKaRa Ex Taq™ Hot Start Version (TaKaRa Bio, Shiga, Japan) was used for amplification. Antimitochondrial peptide nucleic acid (mPNA: 5′-GGCAAGTGTTCTTCGGA-3′) and antiplastid peptide nucleic acid (pPNA: 5′-GGCTCAACCCTGGACAG-3′) were added to reduce the amplification of host DNA (0.25 μM final concentration for each PNA; Fitzpatrick et al., 2018). PCR products were purified using a 1:1 ratio of AMPure XP bead (Beckman Coulter, IN), and quantified using Quant-iT™ PicoGreen^®^ dsDNA detection kits (Invitrogen, San Diego, CA, United States). Final products were used for paired-end read sequencing reactions and sequenced using a MiSeq (2 × 300 bp reads) from the Macrogen Corporation (Seoul, South Korea).

### Bioinformatic Analysis

To investigate the sequence variants at individual nucleotide positions in a gene, amplicon sequence variants (ASVs) of the 16S rRNA genes were calculated using DADA2 (version 1.16), according to the pipeline tutorial 1.16 in R (Callahan et al., 2016).^1^ The latest Silva database (release 138) was used to align and classify the sequences of the 16S rRNA gene (Quast et al., 2013). After classifying the sequences, chloroplast, mitochondrial, and eukaryote sequences were removed from the dataset of the 16S rRNA genes. ASVs that comprised only singletons, doubletons, or tripletons were not further analyzed. Rarefaction curves were constructed using the “rarecurve” function in Vegan (Oksanen et al., 2013). For species-level assignments, we analyzed selected ASVs using BLAST against the NCBI 16S rRNA database. If the blast result of an ASV sequence matched with a single species with >99% similarity, the species name was assigned that ASV. If multiple species were matched with an ASV sequence with the same similarity, no species name was designated to that ASV. The raw sequences and accompanying metadata are available in the Sequence Read Archive of the NCBI under the project accession number PRJNA816676.

All statistical analyses were performed using the R software (version 3.4.0; R Core Team, 2013). A Venn diagram was constructed to show the distribution of ASVs among holobiont components, using the webtool developed by Bioinformatics & Evolutionary Genomics.^2^ Biodiversity indices such as species diversity and richness were calculated using functions in Vegan (Hurlbert, 1971; Oksanen et al., 2013). To normalize the data for diversity indices, the reads were normalized to the lowest number of reads in the “rrarefy” function of Vegan. The Chao1 index and Shannon diversity index were used to estimate microbial diversity and richness for each group. We performed two-way ANOVA and Tukey-HSD test for diversity indices using the “aov” and “tukeyHSD” functions in R. We used non-metric multidimensional scaling (NMDS) analysis with Bray–Curtis distances to order the samples in the prokaryotic community based on their dissimilarity, using the “metaMDS” function in Vegan (Oksanen et al., 2013). The NMDS results were quantitatively evaluated using analysis of similarity (ANOSIM) and permutational multivariate analysis of variance (PERMANOVA) using the “anosim” and “adonis” functions in Vegan, respectively (permutation 999). A phylogenic tree was constructed using a neighbor-joining method in Mega X (Kumar et al., 2018), and bootstrap values were calculated from 1,000 replications.

### Network Analysis

To investigate the relationships among prokaryotes, SparCC networks based on the sampling sites were calculated using the R software package SpiecEasi (Kurtz et al., 2015). To reduce the number of rare ASVs in the data set, only ASVs that met the following thresholds for each given dataset were considered: (1) detected in 25% of samples and (2) a relative proportion of >0.01% for at least one sample. Only positive correlations with SparCC values ≥0.5 were selected for further analyses. The network was visualized using the open-source software Cytoscape 3.5.1 (Shannon et al., 2003). To discover recurrent patterns of site-dependent SparCC networks, site-dependent sparCC networks were combined into one network—the microbial recurrent association network (MRAN)—using the method described by Chun et al. (2019). Network topological parameters, including the average number of neighbors, density, heterogeneity, centralization, average clustering coefficient, characteristic path length, and small-world coefficient, were calculated using the Networkanalyzer plugin in Cytoscape (Assenov et al., 2008; Humphries and Gurney, 2008). Furthermore, Erdős–Rényi random networks with the same number of nodes and links that were randomly distributed were used as a null model for comparison with site-dependent SparCC networks using the Network Randomizer plugin in Cytoscape (Tosadori et al., 2016). In this study, the core taxa was defined as ASV that met the following two requirements: (1) ASVs that appeared in more than 70% samples in a specific holobiont component (in case of root samples, ASVs appeared in more than 90% due to the high microbial diversity of root samples) and (2) ASVs that exhibited repeated correlations of more than four times among five different site-dependent SparCC networks.

### PICRUst2 Analysis

Ecological function profiles for holobiont samples were predicted using PICRUSt2 (Douglas et al., 2020). Abundance data and sequence information for a total of 27,906 prokaryotic ASVs were used as the input file with default options for analysis using picrust2_pipeline.py. To construct non-metric multidimensional scaling (NMDS) based on the functional profiles, a total of 7,107 predicted KEGG Orthologies were selected. Predicted Enzyme Classification numbers were used to assign the proteins to MetaCyc pathways. The abundances of MetaCyc pathways were categorized at superclass levels, and these data were used to construct a heatmap (Caspi et al., 2014).

### Structural Equation Model Analysis

Structural equation modeling (SEM) was used to estimate the relationships among holobiont components, representative bacterial groups, and environmental parameters. To eliminate multicollinear environmental variables, Spearman rank correlation coefficients (*ρ*) were calculated before SEM analysis. The SEM was constructed by using the “sem” function in the Lavaan package (Rosseel et al., 2017). The conceptual model of the hypothetical relationships was that each holobiont component was highly connected and influenced by other holobiont components. For the holobiont components, the first NMDS axis scores were used in the subsequent SEM analysis (Figure 1D). The analysis of the network led to *Rhizobium* cluster, genus *Ralstonia*, and genus *Massilia* being selected as representative groups of rhizospheres, phyllosphere, and dead leaf microbiome, respectively, since these groups of bacteria appeared in the recurrent network and were major components in the specific niches. To add an individual plant as variable, each plant was given a random number using a random number generator in excel. We used five indices of fitness for our model, including (1) the *χ*^2^ test (the model has a good fit when *χ*^2^/df ≤ 5 and 0.05 < *p* ≤ 1.00); (2) the comparative fit index (CFI; 0.95 ≤ CFI < 1.0); (3) the Tucker Lewis Index (TLI; 0.95 ≤ TLI < 1.0); (4) the standardized root mean square residual (SRMR; 0 ≤ SRMR ≤ 0.05); and (5) the root mean square error of approximation (RMSEA; 0 ≤ RMSEA ≤ 0.05; Schermelleh-Engel et al., 2003).

## Results

### Characteristics of Bulk Soil

The characteristics of the bulk soil from each sampling site are summarized in Table 1. Total nitrogen ranged from 0.06% to 0.12%, and available phosphorus ranged from 57.9–147.8 mg/kg. Organic matter ranged from 1.3% to 3.0%. Pairwise *t*-tests demonstrated that total nitrogen, organic matter, cation-exchange capacity, pH, and electrical conductivity were not significantly different between the sampling sites. However, available phosphorus was significantly higher in samples from Gurye than in those from Seosan (*t*-test: *p* < 0.01). About 74% of soil samples were from sandy loam, and the remaining samples were identified as loam and loamy sand.

### Prokaryotic Community Composition

The proportions of chloroplast, mitochondrial, and eukaryotic sequences were in an average of 82.9% and 7.7% in above-ground and belowground samples, respectively. After filtering the raw sequences, an average of 46,000 reads per sample were obtained from bulk soil, lateral root, and primary root samples, while only an average of 2,500 reads per sample were obtained from above-ground samples (Supplementary Table S1). The rarefaction curves indicated that an adequate number of reads was obtained for analysis (Supplementary Figure S1) A total of 27,906 prokaryotic ASVs were identified in the samples. NMDS ordination showed that samples were clustered according to holobiont components (Figure 1D). Both lateral root and primary root samples were separated in bulk soil samples, and the upper part samples were separated from the belowground samples. Dead leaf samples were located between the upper part samples and the belowground samples. Both ANOSIM and PERMANOVA also indicated that prokaryotic communities differed significantly according to the part of the holobiont in which they occurred (ANOSIM, *R* = 0.60, *p* = 0.001 and PERMANOVA, pseudo-*F* = 8.30, *p* = 0.001). However, there was no significant difference by sampling site.

**Figure 2:**
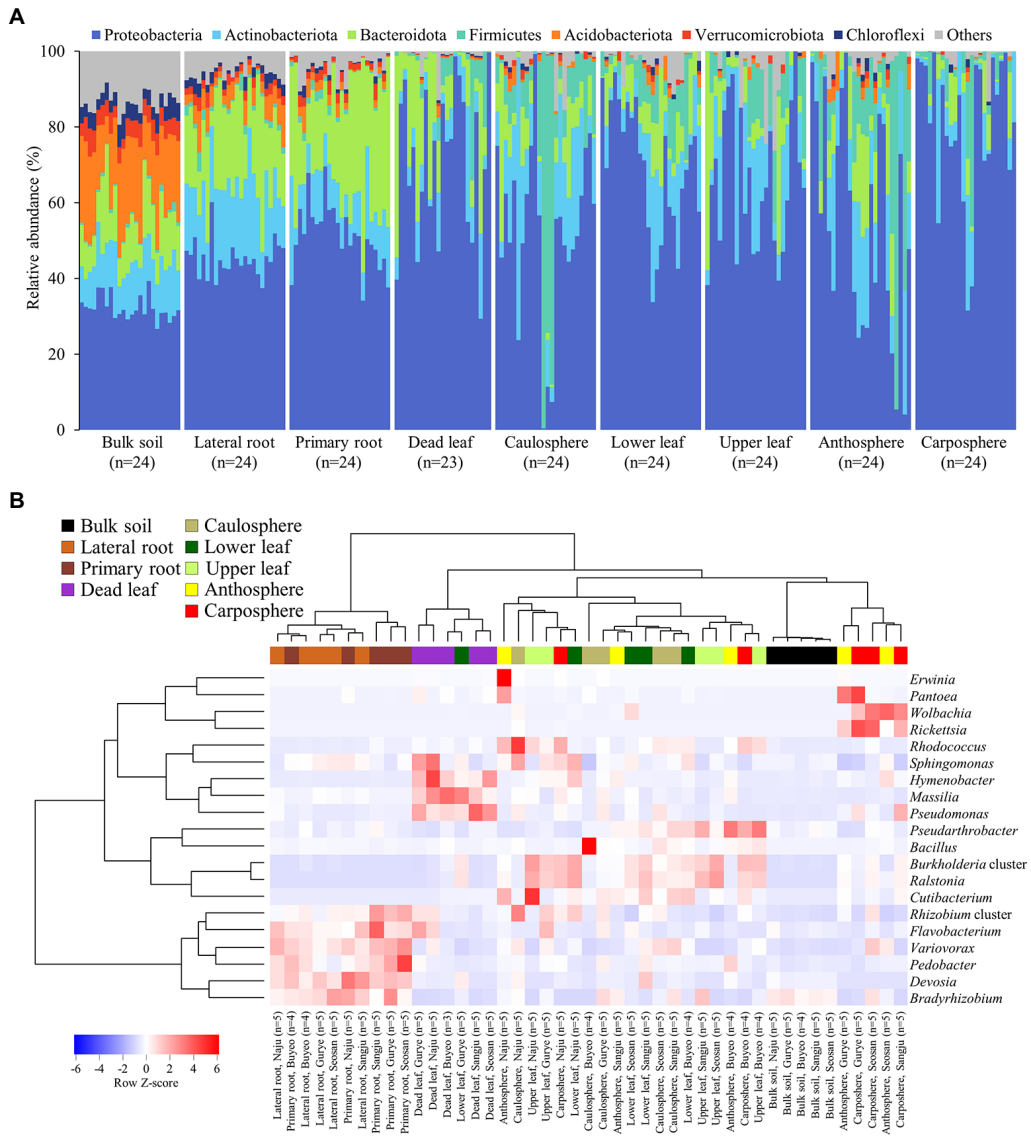
Microbial community composition. **(A)** Relative abundance of prokaryotic community members at the phylum level. **(B)** Heatmap of hierarchical clustering of major bacterial groups at the genus level. Dendrograms were calculated using Euclidean distance and the Ward method. Heatmap color (blue to red) displays the row scaled relative abundance (Row Z-score) of each taxon across all samples. The values of the same holobiont components in the same region are averaged. Samples from different holobiont components are displayed according to the color bar above the heat map.

Forty-nine phyla were identified, and ~60% of the sequences belonged to the phylum *Proteobacteria* (Figure 2A). Acidobacteriota in bulk soil was ~14 times more abundant than other samples, and Bacteroidota in root samples was ~3 times higher than in other samples. Most Firmicute ASVs were found in the upper part of the plant (average relative abundance 9.7%), with only 1.1% found below ground. At the family level, the heatmap showed that most of the samples correctly clustered using hierarchical cluster analysis (Supplementary Figure S2). Even in different sampling sites, bulk soil, lateral root, primary root, and carposphere samples clustered together. At the genus level, *Pseudomonas*, *Massilia*, and *Hymenobacter* were the major groups in dead leaf samples, while a *Ralstonia* and *Burkholderia* cluster (*Burkholderia*, *Caballeronia*, and *Paraburkholderia*) were major groups in healthy leaf samples (Figure 2B). A *Bradyrhizobium*, *Rhizobium* cluster (*Allorhizobium*-*Neorhizobium*, *Pararhizobium*, and *Rhizobium*), and *Devoisa* were enriched in rhizosphere samples. The unclassified *Wolbachia* ASV (ASV20) appeared in 13% of anthosphere and 38% carposphere samples, and did not appear in other components (<4%; maximum relative abundance: 21.9%). Core taxa were designated according to the definition provided in the Materials and Methods section and are summarized in Table 2. Notably, *Ralstonia pickettii* (ASV1) and *Paraburkholderia fungorum* (ASV2) were identified as core taxa in phyllosphere. In addition, *Pseudomonas* (ASV4 and ASV5), *Rhodococcus* (ASV9), and *Massilia* (ASV17, ASV21, and ASV180) were identified as core taxa in the dead leaf microbiome. In this study, only 54 archaeal ASVs were detected, with a relative abundance ranging from 0% to 0.6%. Most archaeal ASVs belonged to the family Nitrososphaeraceae and were found in bulk soil samples.

**Table 2:** Appearance ratio (%) and taxonomy of ASVs according to holobiont components.

ASVs	BS	LR	PR	DL	Cau	UL	LL	Ant	Car	Core taxa	Taxonomy (Class; Genus)
ASV1	13	8	58	**74**	**92**	**100**	**100**	**100**	**100**	O	Gammaproteobacteria; *Ralstonia pickettii*
ASV2	0	0	29	**70**	**92**	**100**	**96**	**71**	**100**	O	Gammaproteobacteria; *Paraburkholderia fungorum*
ASV4	21	17	21	**83**	25	17	8	8	0	O	Gammaproteobacteria; *Pseudomonas viridiflava*
ASV5	4	8	21	**78**	8	8	4	0	4	O	Gammaproteobacteria; *Pseudomonas*
ASV6	**100**	**100**	**100**	52	54	25	17	33	17		Actinobacteria; *Pseudarthrobacter*
ASV7	63	**92**	**75**	52	42	17	13	21	4		Bacilli; *Bacillus*
ASV9	**75**	**83**	**88**	**100**	63	46	29	33	38		Actinobacteria; *Rhodococcus*
ASV10	**96**	**100**	**96**	65	67	25	17	25	17		Bacilli; *Bacillus*
ASV11	4	0	13	65	**83**	**71**	21	42	58	O	Actinobacteria; *Cutibacterium*
ASV12	**100**	**88**	**96**	57	17	29	29	17	21		Actinobacteria; *Pseudarthrobacter*
ASV14	**100**	**100**	**100**	43	33	25	13	25	8		Alphaproteobacteria; *Bradyrhizobium*
ASV17	0	0	0	**74**	17	21	4	8	0	O	Gammaproteobacteria; *Massilia*
ASV18	21	4	25	**91**	46	38	8	13	8	O	Alphaproteobacteria; *Sphingomonas*
ASV21	0	0	4	**74**	8	17	8	4	0	O	Gammaproteobacteria; *Massilia aurea*
ASV29	**96**	**96**	**75**	39	67	17	8	25	13		Bacilli; *Bacillus*
ASV45	13	25	8	**87**	13	21	17	21	17		Alphaproteobacteria; *Rhizobium* cluster
ASV49	50	46	33	**70**	21	21	17	17	0		Bacteroidia; *Pedobacter agri*
ASV58	58	**100**	**88**	4	13	4	0	4	8	O	Alphaproteobacteria; *Caulobacter*
ASV59	**88**	**96**	**88**	9	8	13	0	0	8	O	Alphaproteobacteria; *Devosia*
ASV63	8	8	13	**78**	17	17	0	13	8	O	Actinobacteria; NA
ASV83	46	**92**	**92**	0	13	4	0	0	4	O	Alphaproteobacteria; *Sphingopyxis*
ASV87	**88**	**100**	63	4	21	4	0	0	0		Actinobacteria; *Streptomyces*
ASV89	**100**	**100**	**92**	17	13	0	4	0	0	O	Alphaproteobacteria; *Bradyrhizobium*
ASV96	29	83	**92**	0	4	0	0	0	0	O	Alphaproteobacteria; *Sphingomonas*
ASV123	21	**92**	**71**	4	8	0	0	0	0	O	Alphaproteobacteria; *Mesorhizobium*
ASV129	38	**92**	**71**	4	0	0	0	0	0	O	Actinobacteria; *Streptomyces*
ASV154	**79**	**100**	**92**	4	0	0	0	4	0	O	Alphaproteobacteria; *Rhizobium* cluster
ASV172	4	**96**	**83**	9	8	0	0	0	0	O	Alphaproteobacteria; *Caulobacter*
ASV180	0	0	0	**70**	13	4	0	13	0		Gammaproteobacteria; *Massilia*

BS, bulk soil; LR, lateral root; PR, primary root; DL, dead leaf; Cau, caulosphere; LL, lower leaf; UL, upper leaf; Ant, anthosphere; Car, carposphere. Appearance ratio >70% are highlighted in bold.

**Figure 3:**
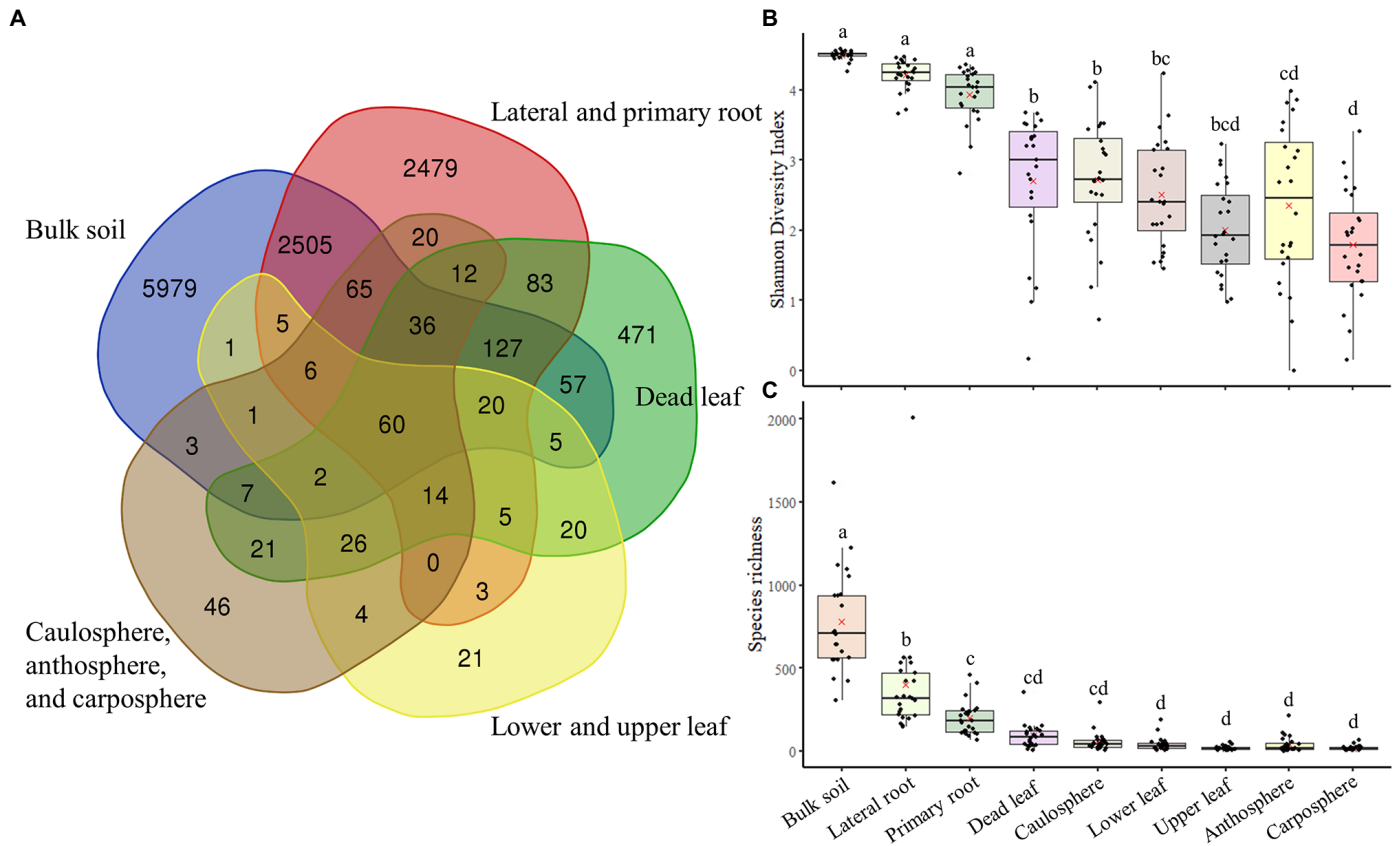
Venn diagram of components, and diversity indices. **(A)** Venn diagram based on ASVs. **(B)** Shannon diversity index. **(C)** The Chao1 index. The letters on the top of the boxes indicates the results of the two-way ANOVA and Tukey-HSD test with the significance threshold of *p* > 0.05.

Analysis using Venn diagrams showed that ~50 of ASVs were only found in bulk soil, while 20% were only found in root samples. Only 71 ASVs were identified as unique to the upper part (caulosphere, anthosphere, and carposphere; Figure 3A). About 500 ASVs were only found in dead leaf samples. Diversity analysis showed that both diversity, as measured by the Shannon diversity index, and richness, as measured by the Chao1 index, were significantly higher in bulk soil samples than in the other samples (*t*-test: *p* < 0.001; Figures 3B,C). Richness significantly decreased in plant upper part samples compared to samples from below ground (*t*-test: *p* < 0.001).

### Site-Dependent SparCC Networks and Recurrent Network

**Figure 4:**
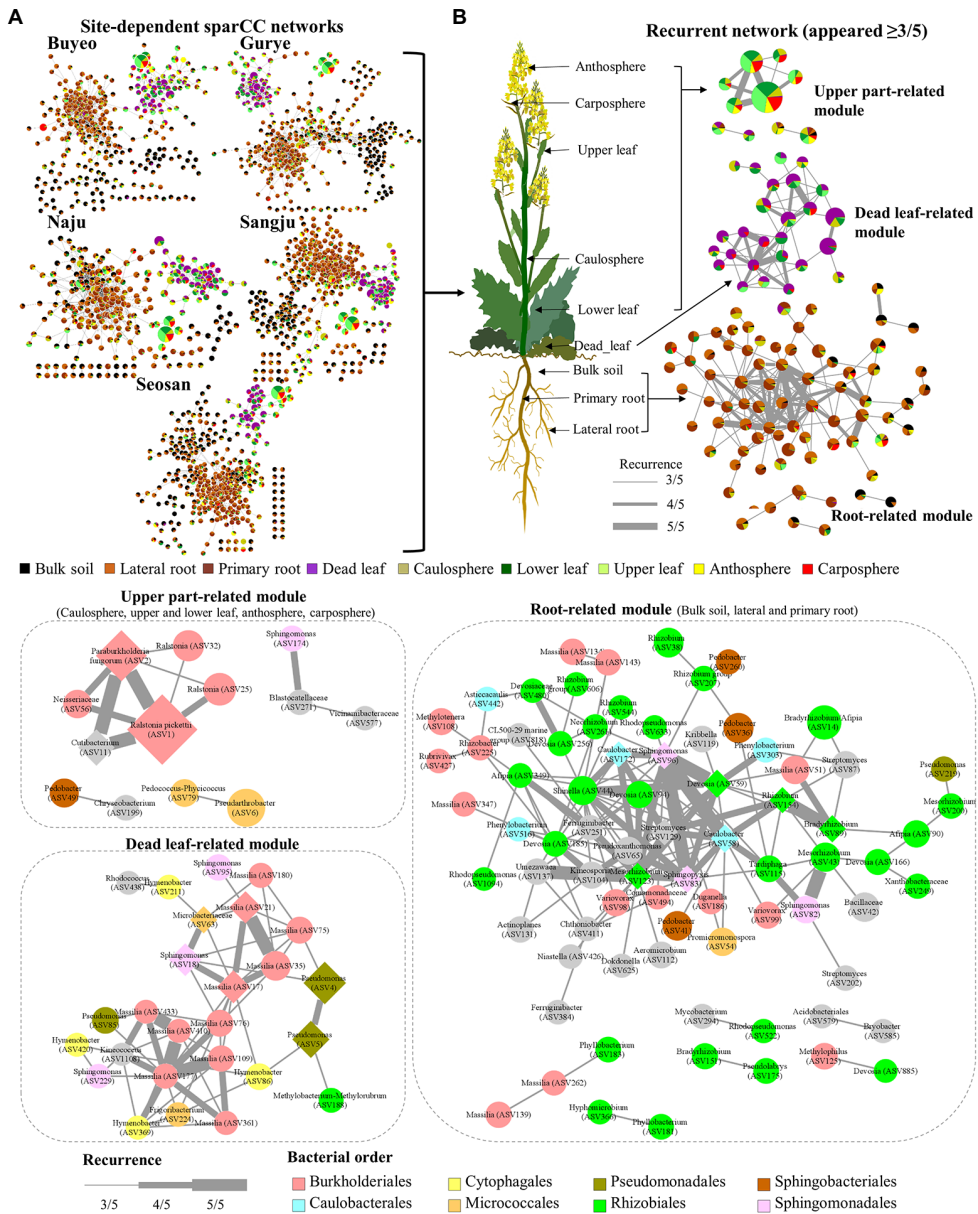
Structure of site-dependent networks and recurrent network. **(A)** Site-dependent networks. The node colors represent the composition of holobiont components in each ASV. The node size represents the average total relative abundance. **(B)** Recurrent network. The thickness of edges represents the recurrence. Diamond nodes represent core taxa.

Five different site-dependent SparCC networks were constructed and visualized (Figure 4A). The topological features of the networks are summarized in Table 3. The networks had 280–370 nodes and 784–1,542 edges. Network heterogeneity, centralization, and average clustering coefficient were higher than those of Erdős-Rényi random networks. Nodes were clustered into three to four groups, corresponding to root, bulk soil, dead leaf, or upper part, in each site-dependent SparCC network. To overcome some of the limitations of conventional network analysis, five different networks were merged into a single network, called a recurrent network, and edges found in at least three different networks were incorporated (appeared ≥3/5; Figure 4B). The recurrent network consisted of 116 nodes and 211 edges, and was divided into three major modules: a root-related module, a dead leaf-related module, and an upper part-related module (Table 3). Bulk soil-related modules were apparent in the site-dependent SparCC networks, but not in the recurrent network. Taxonomically, three ASVs assigned to the genus *Ralstonia* were major components in the upper part-related module, and ASVs assigned to the genera *Massilia* and *Hymenobacter* were major components in the dead leaf-related module (Figure 2B). The major *Ralstonia* ASV (ASV1) was identified as *R. pickettii* (sequence similarity 100%) and other *Ralstonia* ASVs showed lower sequence similarity (97.8%–98.3%) with other isolated strains. *Ralstonia pickettii* (ASV1) had high recurrent connections between *Cutibacterium acnes* (sequence similarity 100%) and *Paraburkholderia fungorum* (sequence similarity 100%) in the upper part-related module. Eleven different *Massilia* ASVs were observed, and only one *Massilia* ASV (ASV17) was identified as *Massilia aurea* (sequence similarity 100%; Supplementary Figure S3), while the other *Massilia* ASVs had lower sequence similarity (98.5%–99.7%) with other isolated strains. *Massilia* spp. had a large number of recurrent connections with *Hymenobacter*, *Sphingomonas*, *Microbacteriaceae*, and *Kinecoccus* in the dead leaf-related module. ASVs belonging to Rhizobiales, especially *Devosia*, were major components in the root-related module. A large number of recurrent connections were observed among *Caulobacter*, *Sphingomonas*, *Sphingopyxis*, and *Mesorhizobium* in the root-related module. Six different *Massilia* ASVs were also found in the root-related module; however, these ASVs were identified as a group of *Massilia atriviolacea* (sequence similarity 98.6%–100%). The appearance ratio also supported the results of the recurrent network (Table 2). For instance, *R. pickettii* (ASV1) and *Paraburkholderia fungorum* (ASV2) were found in over 95% of the phyllosphere samples. In the dead leaf microbiome, *Rhodococcus* (ASV9) and *Sphingomonas* (ASV18) appeared in more than 90% of samples.

**Table 3:** Topological characteristics of microbial networks.

Topological characteristics	Buyeo	Gurye	Naju	Sangju	Seosan	MRAN (3/5)
Nodes	355	370	280	328	340	116
Edges	1,542	1,434	1,044	992	784	211
Diameter	14	13	9	8	8	8
Average number of neighbors	10.65	8.5	8.92	6.86	5.03	4.16
Network density	0.04	0.03	0.05	0.03	0.02	0.07
Network heterogeneity	1.06	1.02	0.97	0.93	0.92	0.97
Network heterogeneity, random	0.35	0.36	0.38	0.41	0.48	0.44
Centralization	0.16	0.12	0.18	0.11	0.09	0.23
Centralization, random	0.03	0.02	0.03	0.03	0.02	0.05
Average clustering coefficient (C)	0.42	0.43	0.34	0.43	0.34	0.16
Clustering coefficient, random (C_r_)	0.03	0.02	0.03	0.02	0.02	0.02
Characteristic path length (L)	5.2	4.9	3.3	5.4	5.3	3.27
Characteristic path length, random (L_r_)	3	3.1	3	3.4	3.9	3.8
C/C_r_	14	21.5	11.3	21.5	17.0	8.0
L/L_r_	1.7	1.6	1.1	1.6	1.4	0.8
Small-world coefficient (SW)	8.1	13.6	10.3	13.5	12.5	9.3

### Ecological Function Analysis by Using PICRUSt2

**Figure 5:**
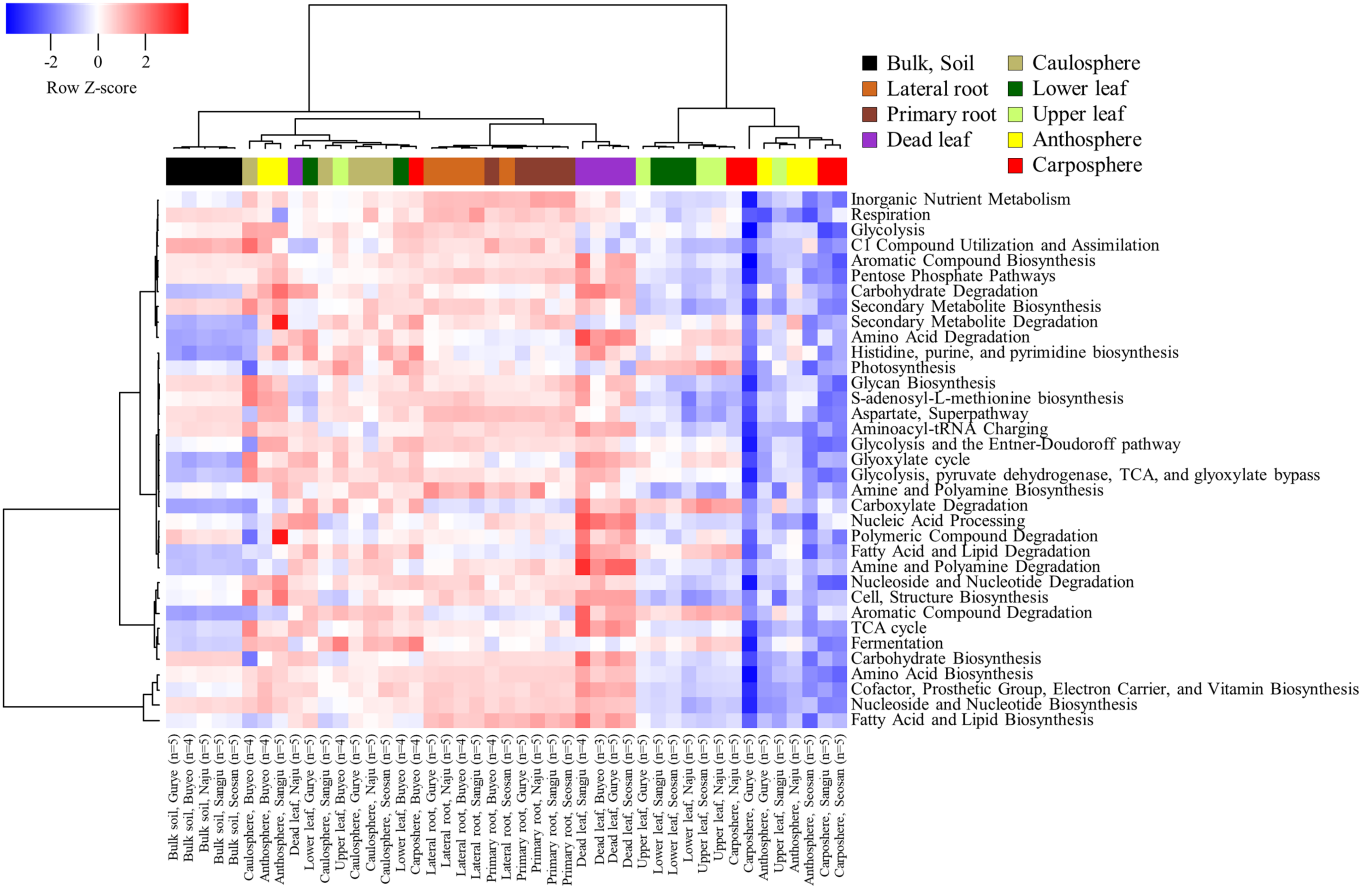
Heatmap of hierarchical clustering of functional groups predicted by Picrust2 analysis.

A total of 7,902 KEGG Orthologies and 441 MetaCyc pathways were predicted in the samples by using PICRUSt2 analysis. The NMDS ordination based on KEGG Orthologies showed that samples were clustered according to the holobiont components, especially in the bulk soil, rhizosphere, and dead leaf samples (Supplementary Figure S3). The predicted functional pathways were consistent for each category in the soil, rhizosphere, and dead leaf samples (Figure 5). Most of the predicted pathways were more expressed in the rhizosphere and dead leaf samples. For instance, pathways related to the degradation of carbohydrates, amino acids, amines, carboxylate, polyamines, aromatic compounds, fatty acids, lipids, and polymeric compounds were more active in dead leaf samples. Pathways involved in inorganic nutrient metabolism and the degradation of secondary metabolites were more active in rhizosphere samples than in bulk soil samples, while functions related to the utilization of C1 compounds and the assimilation and degradation of polymeric compounds were more active in bulk soil samples. In the phyllosphere samples, pathways related to photosynthesis, carboxylate degradation, fermentation, and degradation of aromatic compounds were predicted to be major pathways.

### Structural Equation Model

**Figure 6:**
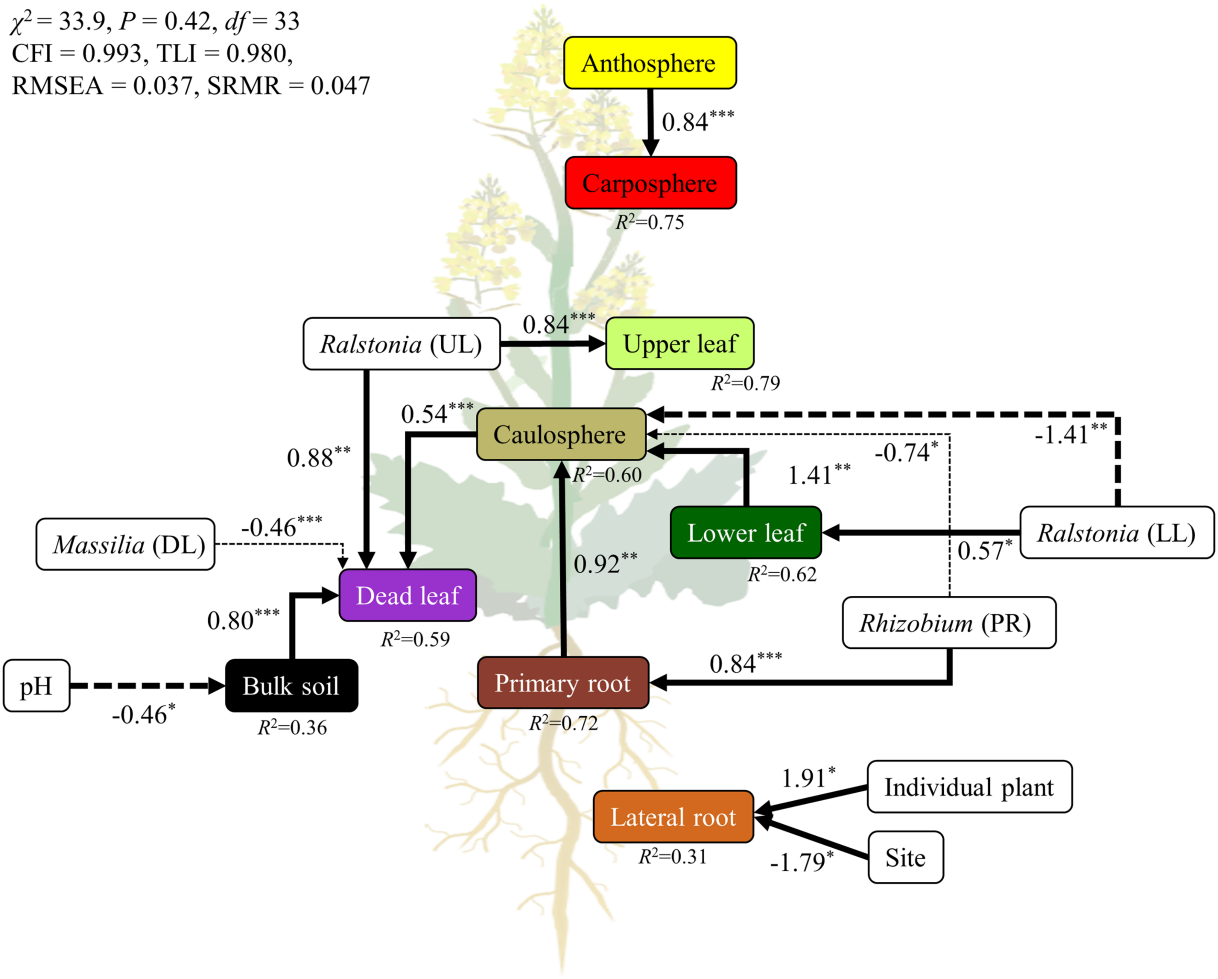
Structural equation model (SEM) showing the relationships among holobiont components, environmental factors, and representatives groups of bacteria. The solid arrows represent the positive effects, while dotted arrows represent the negative effects. The width of the arrows indicates the strength of the effect. ^***^p < 0.001; ^**^p < 0.01; ^*^p < 0.05.

A SEM was constructed to test a hypothetical link between holobiont components, environmental parameters, and representative bacterial groups (Figure 6). We compared several candidate models, and selected the best fitting model. The final SEM had a *χ*^2^ test statistic of 33.9 with 33 degrees of freedom, an RMSEA of 0.037, CFI of 0.993, TLI of 0.980, and SRMR of 0.047 (Figure 6). These indices of fit indicated that the model operated within the acceptable limits, and had a good fit for its purpose. Overall, the model explained 59%–75% of the variance in above-ground holobiont components and the primary root microbiome, while explaining 31%–36% of the variance in bulk soil and the lateral root microbiome. The model identified pH as a major driver of the bulk soil microbiome. Representative groups of bacteria from the recurrent network significantly affected the microbial community of the holobiont. *Ralstonia*, *Rhizobium*, and *Massilia* were the strongest drivers of the microbial community in the phyllosphere, rhizosphere, and bulk soil microbiome, respectively (Figure 6). However, the lateral root microbiome was only affected by individual plant and site. The relationships among the holobiont components showed that the caulosphere was strongly influenced by the primary root and lower leaf microbiome, and in the case of dead leaves, the soil and the caulosphere were direct drivers. The carposphere was found to be significantly affected by the anthosphere, and no factors affecting the anthosphere were found.

## Discussion

Plants always coexist and interact with various microorganisms in natural ecosystems, and the importance of the microbiome in different holobiont components is being increasingly realized (Vandenkoornhuyse et al., 2015; Sánchez-Cañizares et al., 2017). Many studies have been performed to investigate the relationship between *B. napus* and its microbiome (Taye et al., 2019; Bell et al., 2021; Morales Moreira et al., 2021). However, most of this research has been conducted on plants grown in cultivated fields, rather than in natural ecosystems, such as grassland. A variety of plants and microorganisms coexist and show more intensive interactions in the natural ecosystem than in cultivated fields (Chen et al., 2015; Arunkumar et al., 2019). To our knowledge, few studies focused on the microbial diversity of dead leaves, especially on the fungal diversity on dead leaves of woody plants, in natural ecosystems (U’Ren and Arnold, 2016; Guerreiro et al., 2018). Almost no studies focused on the bacterial community on dead leaves of herbaceous plants. We observed significant differences in microbial community structure and diversity among the different types of holobiont components (Figures 1D, 3B), indicating that the plant holobiont components are a major selective force that shapes the structure of the plant-associated microbiome. Cregger et al. (2018) and Trivedi et al. (2020) showed that prokaryotic diversity increased from leaf, to caulosphere, to rhizosphere, to bulk soil habitats. Consistently, a rapid decrease in diversity from bulk soil to rhizosphere and then to plant phyllosphere was observed in this study (Figure 3B). Higher values of diversity indexes were observed in the dead leaf microbiome than in the other phyllosphere components. The diversity of the leaf microbiome has been reported to be relatively constant, with a slight increase over time (Wagner et al., 2016). However, when defense mechanisms and metabolic activity decrease in old leaves, a rapid shift in the microbial community occurs in the leaf microbiome (Mora-Gómez et al., 2016). We found that ASVs belonging to *Massilia* were abundant in the dead leaf microbiome, and *Ralstonia* ASVs were a major group in the healthy leaf microbiome. The two groups showed different patterns of co-occurrence (Figure 2B). *Massilia* is a well-known rhizospheric bacterium, which plays a key role in the succession of microbes in the early stages of the rhizosphere (Ofek et al., 2012). *Massilia* has also been identified as a network hub, and contributes to forming distinct microbiomes according to the host plants (Lewin et al., 2021). In this study, a phylogenic tree of the top 20 ASVs of *Massilia* indicated that distinct clusters of the dead leaf- or rhizosphere-related *Massilia* spp. existed (Supplementary Figure S3). *Massilia* correlated with *Sphingomonas*, *Hymenobacter*, *Kineococcus*, and *Pseudomonas* in the dead leaf microbiome (Figure 4B). Herrmann et al. (2021) showed that harsh environmental conditions, such as UV radiation, desiccation, precipitation, and low nutrient conditions, appear to be the driving force of the dominance of *Massilia*, *Hymenobacter*, and *Kineococcus* in the phyllosphere. These results suggested that unique *Massilia* ASVs are present in different parts of the holobiont. These microbes could be more adaptable for the aging of plant leaves and lead the transition of the whole microbial community structures of dead leaf microbiome in *B. napus* (Figure 6).

Functional profiles suggested that the dead leaf microbiome was involved in various degradation processes (Figure 5). The quality of the initial litter, and environmental variables, are critical factors that influence microbial degradation (Bray et al., 2012; Bani et al., 2018). For example, insecticidal proteins produced by a genetically modified plant remain in the litter and affect the surrounding biome, reducing the leaf decomposition rate compared to that of non-transgenic plants (Donegan et al., 1997; Flores et al., 2005). Taken together, these results indicate that the effect of insecticidal proteins on specific *Massilia* sp. should be considered in the environmental risk assessment of genetically modified plants.

Unlike other plant holobiont components, the flower is a transient microhabitat that interacts with a variety of organisms, such as pollinators and nectar robbers (Vannette, 2020). However, in this study we did not find any recurrent patterns related to the anthosphere. An unclassified Corynebacteriales ASV, ASV68, appeared in 40% of anthosphere samples, but did not appear in other components. The BLAST result showed that ASV68 had 100% similarity with the uncultured bacterial clones KF600023, KF599927, KF599867, and KF599666, which were obtained from beebread produced by honey bees (*Apis mellifera*; Anderson et al., 2013). *Gilliamella* sp. (ASV31), known to be a honey bee gut symbiont, was also found in up to 86% of anthosphere samples from Gurye (Zheng et al., 2016). These results suggest that these bacteria were derived from honey bees and could be used as indicator species for the identification of visitation by honey bees. In this study, five different ASVs belonging to the genus *Wolbachia* were found in the phyllosphere. The genus *Wolbachia* is one of the most dominant arthropod endosymbionts, and some species belonging to the genus *Wolbachia* could be transferred to the plants from the host during physical contact between arthropod and plant (Sanaei et al., 2021). *Wolbachia* can persist within or on the surface of the plant for at least 50 days, and can be transferred to new hosts (Li et al., 2017). Flowers can also be used as an entry route for plant pathogenic bacteria. For example, *Erwinia persicina* (ASV13), known to be a phytopathogenic bacterium (Zhang and Nan, 2014), was predominant in three anthosphere samples (16%–99%) from Naju (Figure 2B). These results suggest that plant holobiont analysis is essential to understanding these complex interactions in the natural ecosystem, and plants serve as an ecological platform which connects the insect and plant microbiomes (Liu et al., 2019).

Since soil environmental conditions are highly heterogeneous, soil microbiomes can have a wide range of unique microbial assemblages (Fierer, 2017). However, plant roots and related microbes interact with the surrounding soil microbes to form a relatively constant rhizosphere, in contrast to the bulk soil microbiome (Philippot et al., 2013). Consistent with previous results (Praeg and Illmer, 2020), we found that the phylum Bacteroidetes was significantly more abundant in rhizosphere soils than in bulk soil (Figure 2A). Interactions within the bulk soil microbiome were evident in the site-specific networks (Figure 4A), but not in the recurrent network (Figure 4B). This result suggested that the interactions within the bulk soil microbiome were highly site-specific; however, the interactions within rhizosphere microbes are relatively consistent.

Plant species harbor unique, persistent, core and hub microbes, and these microbes provide useful information about plant fitness and productivity (Trivedi et al., 2020). Taye et al. (2019) and Floc’h et al. (2020) revealed that there are some core microbes, such as *Arthrobacter*, *Bradyrhizobium*, *Pseudarthrobacter*, and *Stenotrophomonas*, in the rhizosphere of *B. napus* grown in the field. Consistently, *Pseudarthrobacter* (ASV6 and ASV12) and *Bradyrhizobium* (ASV14 and ASV89) were also appeared more than 70% in the rhizosphere samples of feral *B. napus* (Figure 4; Table 2). ASV172 (*Caulobacter* sp.) and ASV186 (*Duganella* sp.) appeared in more than 80% of the rhizosphere samples and <4% of the bulk soil samples. Due to the limitations of the sampling methods, bulk soils usually contain soil particles separated from the roots. Therefore, these results implied that these species could be specialists in the root endosphere, but the origin of these species remains unclear.

All components of a plant are interconnected, and the movement of microbes through the inside and outside of a plant plays an important role in the formation and maintenance of plant holobionts (Kumar et al., 2020). The stem connects the root and the leaf, and most microbial movement is expected to occur through the stem (Zinniel et al., 2002). In this study, the model showed that the microbial structure of different holobiont components influenced the different parts of the holobiont (Figure 6). For example, the caulosphere was influenced by the rhizosphere, and affected the dead leaf microbiome. Representative bacterial groups, such as *Ralstonia*, *Massilia*, and *Rhizobium*, were also identified as major drivers of the formations of holobionts. However, ~37% of ASVs were only found in a single holobiont component, while 2.3% of ASVs were found in at least four different holobiont components, an observation which suggests that even though bacterial strains can translocate to different components of the holobiont, most of them prefer to specialize in a specific habitat. Therefore, the above representative bacterial groups appear to be consistent across regions, and these microbes contribute to build the complex holobiont through the caulosphere.

Alkanes, fatty acids, and phenolic compounds are the major phytochemical groups in the leaves of *Brassica* species (Velasco et al., 2011; Tassone et al., 2016). Some phenolic compounds and fatty acids have antibacterial activity, and play roles in plant-bacterium signaling in the plant microbiome (McGaw et al., 2002; Pereira et al., 2007; Mandal et al., 2010). The composition of phytochemical compounds is thus one of the major driving forces in the succession of the leaf microbiome. The microbiome of the phyllosphere possessed metabolic abilities related to the fermentation and degradation of carboxylate, fatty acids, lipids, and aromatic compounds (Figure 5). *Ralstonia pickettii* (ASV1), the most abundant species in the phyllosphere in this study, prefers an oligotrophic environment, and metabolizes various compounds as energy and carbon sources using multi-enzyme pathways such as the Tbu pathway (Mandal et al., 2010). *Paraburkholderia fungorum*, the second most abundant species identified in the phyllosphere in this study, is known to be a multifunctional plant probiotic, increasing the productivity and quality of strawberry fruits, and possessing antagonistic activities against major phytopathogens (Rahman et al., 2018). The consistent co-occurrence of *R. pickettii* and *Paraburkholderia fungorum* could be the foundation of the maintenance and activity of the leaf microbiome of *B. napus* (Figure 4).

## Conclusion

We found that the microbial community structure and diversity were significantly different in different components of the plant holobiont. The microbial structure of the different holobiont components influenced the different parts of the holobiont. Despite the long distance between sampling sites, each individual holobiont and its core microbes (mostly the representative bacterial groups) were relatively constant across the region, indicating that the plant species itself, directly or indirectly *via* its core microbes, determine their own microbiome. Representative bacterial groups, such as *Ralstonia*, *Massilia*, and *Rhizobium* clusters, were one of the major drivers of the formation of the holobionts of *B. napus*. The anthosphere possessed distinct microbial groups, which posed as the ecological platform of arthropod-plant-bacteria interactions in natural ecosystem. To summarize, each holobiont component was organically connected, and microbes, especially the core microbes, in plant holobiont exhibited holobiont-specific recurrent interaction patterns that are essential for the survival and coexistence of plant microbes in natural ecosystems.

## Data Availability Statement

The datasets presented in this study can be found in online repositories. The names of the repository/repositories and accession number(s) can be found at: https://www.ncbi.nlm.nih.gov/, PRJNA816676.

## Author Contributions

S-JC analyzed the data, wrote the manuscript, conceived and supervised the study. S-HY performed the field sampling. YC verified the analytical methods and analyzed the data. JL conceived and supervised the study. All authors contributed to the article and approved the submitted version.

## Funding

This research was supported by the National Institute of Ecology (NIE) funded by the Ministry of Environment (MOE) of the South Korea (NIE-A-2022-10).

## Conflict of Interest

The authors declare that the research was conducted in the absence of any commercial or financial relationships that could be construed as a potential conflict of interest.

## Publisher’s Note

All claims expressed in this article are solely those of the authors and do not necessarily represent those of their affiliated organizations, or those of the publisher, the editors and the reviewers. Any product that may be evaluated in this article, or claim that may be made by its manufacturer, is not guaranteed or endorsed by the publisher.

## Supplementary Material

The Supplementary Material for this article can be found online at: https://www.frontiersin.org/articles/10.3389/fmicb.2022.920759/full#supplementary-material

## Footnotes

^1^https://benjjneb.github.io/dada2/tutorial.html (Accessed: November 2021).

^2^ https://bioinformatics.psb.ugent.be/webtools/Venn

## Abbreviations

ANOSIM: Analysis of similarity
ASV: Amplicon sequence variants
EC: Enzyme Classification
NIAST: National Institute of Agricultural Science and Technology
NMDS: Non-metric multidimensional scaling
PERMANOVA: Permutational multivariate analysis of variance
RMSEA: Root mean square error of approximation
SEM: Structural equation model
SRA: Sequence Read Archive
SRMR: Standardized root mean square residual
