# Supplementary material for: Organic Connection of Holobiont Components and the Essential Roles of Core Microbes in the Holobiont Formation of Feral Brassica napus
Source: Front Microbiol. 2022 Jul 8;13:920759. doi: 10.3389/fmicb.2022.920759 (PMC9305074; doi:10.3389/fmicb.2022.920759)
Supplement: Supplementary file 1 [file Data_Sheet_1.docx]

**Supplementary material**

**Organic connection of holobiont components and the essential roles of core microbes in the holobiont formation of feral *Brassica napus***

Seong-Jun Chun^a, †, *^, Yingshun Cui^b, †^, Su-Hyang Yoo^a^, Jung Ro Lee^a^

^a^ LMO Team, National Institute of Ecology, 1210 Geumgang-ro, Maseo-myeon, Seocheon 33657, Republic of Korea

^b^ Cell Factory Research Center, Korea Research Institute of Bioscience and Biotechnology, 125 Gwahak-ro, Yuseong-gu, Daejeon 34141, Republic of Korea

* Corresponding author: Seong-Jun Chun, Tel: +82 41 950 5827; Fax: +82 41 950 6156; E-mail: sjchun@nie.re.kr

[^†^] These authors contributed equally to the work.

**Table S1.** Numbers of raw read and filtered reads.

| **Holobionts** | **Plant label** | **Sites** | **Raw reads** | **After filtering** | **Numbers of ASVs** |
| --- | --- | --- | --- | --- | --- |
| Bulk soil | B121 | Naju | 29668 | 27943 | 1010 |
| Bulk soil | B122 | Naju | 55190 | 53239 | 1795 |
| Bulk soil | B123 | Naju | 57514 | 55381 | 1961 |
| Bulk soil | B124 | Naju | 54426 | 50529 | 1671 |
| Bulk soil | B125 | Naju | 53879 | 49119 | 1913 |
| Bulk soil | B126 | Gurye | 68145 | 64854 | 2462 |
| Bulk soil | B127 | Gurye | 61942 | 56812 | 1877 |
| Bulk soil | B128 | Gurye | 65991 | 59026 | 1974 |
| Bulk soil | B129 | Gurye | 61654 | 58436 | 2068 |
| Bulk soil | B130 | Gurye | 60444 | 57312 | 2119 |
| Bulk soil | B131 | Sangju | 45738 | 43250 | 1528 |
| Bulk soil | B132 | Sangju | 45897 | 44550 | 1704 |
| Bulk soil | B133 | Sangju | 49842 | 48360 | 1855 |
| Bulk soil | B134 | Sangju | 51778 | 50398 | 2040 |
| Bulk soil | B135 | Sangju | 65052 | 63008 | 2386 |
| Bulk soil | B136 | Seosan | 60276 | 58431 | 2272 |
| Bulk soil | B137 | Seosan | 51668 | 50021 | 2230 |
| Bulk soil | B138 | Seosan | 62684 | 58913 | 2159 |
| Bulk soil | B139 | Seosan | 48359 | 46804 | 2059 |
| Bulk soil | B140 | Seosan | 49743 | 48388 | 2182 |
| Bulk soil | B141 | Buyeo | 44495 | 39488 | 1664 |
| Bulk soil | B142 | Buyeo | 65577 | 63522 | 2412 |
| Bulk soil | B143 | Buyeo | 52468 | 51117 | 2307 |
| Bulk soil | B144 | Buyeo | 47327 | 45469 | 1940 |
| Lateral root | B121 | Naju | 39925 | 38979 | 1157 |
| Lateral root | B122 | Naju | 39223 | 37823 | 827 |
| Lateral root | B123 | Naju | 35540 | 35024 | 872 |
| Lateral root | B124 | Naju | 39490 | 38267 | 777 |
| Lateral root | B125 | Naju | 41312 | 40236 | 842 |
| Lateral root | B126 | Gurye | 43236 | 42386 | 1025 |
| Lateral root | B127 | Gurye | 47492 | 46251 | 1061 |
| Lateral root | B128 | Gurye | 42392 | 39010 | 1028 |
| Lateral root | B129 | Gurye | 38627 | 36422 | 1286 |
| Lateral root | B130 | Gurye | 31193 | 29477 | 1031 |
| Lateral root | B131 | Sangju | 28755 | 27956 | 1188 |
| Lateral root | B132 | Sangju | 16965 | 16683 | 341 |
| Lateral root | B133 | Sangju | 32585 | 31889 | 1034 |
| Lateral root | B134 | Sangju | 43056 | 42063 | 613 |
| Lateral root | B135 | Sangju | 40261 | 38319 | 842 |
| Lateral root | B136 | Seosan | 55595 | 54338 | 1799 |
| Lateral root | B137 | Seosan | 38269 | 37141 | 1304 |
| Lateral root | B138 | Seosan | 38659 | 37483 | 967 |
| Lateral root | B139 | Seosan | 37276 | 36308 | 1069 |
| Lateral root | B140 | Seosan | 38462 | 36757 | 1177 |
| Lateral root | B141 | Buyeo | 42011 | 39981 | 986 |
| Lateral root | B142 | Buyeo | 51670 | 49155 | 764 |
| Lateral root | B143 | Buyeo | 43438 | 42464 | 967 |
| Lateral root | B144 | Buyeo | 75148 | 72544 | 1192 |
| Upper leaf | B121 | Naju | 57748 | 193 | 9 |
| Upper leaf | B122 | Naju | 57781 | 214 | 9 |
| Upper leaf | B123 | Naju | 51505 | 295 | 9 |
| Upper leaf | B124 | Naju | 60195 | 147 | 10 |
| Upper leaf | B125 | Naju | 59838 | 515 | 35 |
| Upper leaf | B126 | Gurye | 54688 | 6093 | 35 |
| Upper leaf | B127 | Gurye | 58393 | 173 | 18 |
| Upper leaf | B128 | Gurye | 44584 | 828 | 41 |
| Upper leaf | B129 | Gurye | 50258 | 656 | 49 |
| Upper leaf | B130 | Gurye | 53912 | 105 | 10 |
| Upper leaf | B131 | Sangju | 46057 | 104 | 6 |
| Upper leaf | B132 | Sangju | 42944 | 185 | 13 |
| Upper leaf | B133 | Sangju | 60118 | 102 | 4 |
| Upper leaf | B134 | Sangju | 55678 | 175 | 19 |
| Upper leaf | B135 | Sangju | 52554 | 103 | 10 |
| Upper leaf | B136 | Seosan | 80062 | 259 | 14 |
| Upper leaf | B137 | Seosan | 65608 | 121 | 5 |
| Upper leaf | B138 | Seosan | 54679 | 120 | 4 |
| Upper leaf | B139 | Seosan | 50177 | 562 | 34 |
| Upper leaf | B140 | Seosan | 47710 | 183 | 17 |
| Upper leaf | B141 | Buyeo | 42995 | 358 | 24 |
| Upper leaf | B142 | Buyeo | 49318 | 463 | 33 |
| Upper leaf | B143 | Buyeo | 48373 | 280 | 22 |
| Upper leaf | B144 | Buyeo | 51340 | 187 | 13 |
| Lower leaf | B121 | Naju | 73550 | 249 | 14 |
| Lower leaf | B122 | Naju | 51594 | 411 | 13 |
| Lower leaf | B123 | Naju | 46026 | 807 | 32 |
| Lower leaf | B124 | Naju | 60063 | 111 | 8 |
| Lower leaf | B125 | Naju | 62592 | 5148 | 86 |
| Lower leaf | B126 | Gurye | 38544 | 10921 | 26 |
| Lower leaf | B127 | Gurye | 44212 | 1371 | 82 |
| Lower leaf | B128 | Gurye | 59828 | 3227 | 68 |
| Lower leaf | B129 | Gurye | 58422 | 623 | 22 |
| Lower leaf | B130 | Gurye | 60221 | 122 | 8 |
| Lower leaf | B131 | Sangju | 47781 | 706 | 38 |
| Lower leaf | B132 | Sangju | 28656 | 259 | 13 |
| Lower leaf | B133 | Sangju | 43605 | 2201 | 84 |
| Lower leaf | B134 | Sangju | 57515 | 315 | 25 |
| Lower leaf | B135 | Sangju | 45149 | 548 | 35 |
| Lower leaf | B136 | Seosan | 58939 | 576 | 32 |
| Lower leaf | B137 | Seosan | 48687 | 1129 | 27 |
| Lower leaf | B138 | Seosan | 55473 | 898 | 37 |
| Lower leaf | B139 | Seosan | 49330 | 303 | 15 |
| Lower leaf | B140 | Seosan | 51184 | 672 | 50 |
| Lower leaf | B141 | Buyeo | 46171 | 1237 | 55 |
| Lower leaf | B142 | Buyeo | 45079 | 641 | 59 |
| Lower leaf | B143 | Buyeo | 48252 | 10079 | 519 |
| Lower leaf | B144 | Buyeo | 54302 | 3563 | 195 |
| Primary root | B121 | Naju | 60489 | 52695 | 750 |
| Primary root | B122 | Naju | 52784 | 40353 | 890 |
| Primary root | B123 | Naju | 39400 | 32833 | 923 |
| Primary root | B124 | Naju | 67440 | 56110 | 447 |
| Primary root | B125 | Naju | 58777 | 52387 | 1061 |
| Primary root | B126 | Gurye | 59411 | 48079 | 489 |
| Primary root | B127 | Gurye | 45380 | 44393 | 330 |
| Primary root | B128 | Gurye | 55682 | 45109 | 902 |
| Primary root | B129 | Gurye | 50523 | 44627 | 1303 |
| Primary root | B130 | Gurye | 46701 | 27168 | 543 |
| Primary root | B131 | Sangju | 62697 | 58192 | 517 |
| Primary root | B132 | Sangju | 48394 | 45120 | 640 |
| Primary root | B133 | Sangju | 50897 | 48072 | 623 |
| Primary root | B134 | Sangju | 31277 | 30722 | 200 |
| Primary root | B135 | Sangju | 58704 | 43060 | 455 |
| Primary root | B136 | Seosan | 63032 | 56335 | 641 |
| Primary root | B137 | Seosan | 76540 | 68623 | 1392 |
| Primary root | B138 | Seosan | 71019 | 63305 | 724 |
| Primary root | B139 | Seosan | 50804 | 45434 | 803 |
| Primary root | B140 | Seosan | 51924 | 41024 | 347 |
| Primary root | B141 | Buyeo | 67171 | 61949 | 526 |
| Primary root | B142 | Buyeo | 73749 | 54997 | 1005 |
| Primary root | B143 | Buyeo | 64630 | 58449 | 734 |
| Primary root | B144 | Buyeo | 49958 | 39340 | 814 |
| Dead leaf | B121 | Naju | 75597 | 60488 | 324 |
| Dead leaf | B122 | Naju | 56481 | 37045 | 535 |
| Dead leaf | B123 | Naju | 34135 | 33137 | 111 |
| Dead leaf | B124 | Naju | 39941 | 39644 | 145 |
| Dead leaf | B125 | Naju | 108629 | 105106 | 371 |
| Dead leaf | B126 | Gurye | 43814 | 42999 | 166 |
| Dead leaf | B127 | Gurye | 42405 | 41656 | 144 |
| Dead leaf | B128 | Gurye | 47492 | 46551 | 231 |
| Dead leaf | B129 | Gurye | 52024 | 51508 | 258 |
| Dead leaf | B130 | Gurye | 42396 | 40948 | 258 |
| Dead leaf | B131 | Sangju | 50243 | 49424 | 58 |
| Dead leaf | B132 | Sangju | 43417 | 42669 | 42 |
| Dead leaf | B134 | Sangju | 51351 | 49790 | 190 |
| Dead leaf | B135 | Sangju | 51966 | 49621 | 419 |
| Dead leaf | B136 | Seosan | 58081 | 27263 | 318 |
| Dead leaf | B137 | Seosan | 45796 | 9248 | 133 |
| Dead leaf | B138 | Seosan | 38706 | 13949 | 286 |
| Dead leaf | B139 | Seosan | 41987 | 41502 | 117 |
| Dead leaf | B140 | Seosan | 49513 | 48690 | 33 |
| Dead leaf | B141 | Buyeo | 49650 | 11535 | 339 |
| Dead leaf | B142 | Buyeo | 43237 | 32253 | 285 |
| Dead leaf | B143 | Buyeo | 46660 | 32889 | 355 |
| Dead leaf | B144 | Buyeo | 62408 | 43457 | 463 |
| Caulosphere | B121 | Naju | 56012 | 4687 | 26 |
| Caulosphere | B122 | Naju | 51862 | 2232 | 164 |
| Caulosphere | B123 | Naju | 43571 | 686 | 54 |
| Caulosphere | B124 | Naju | 54222 | 1697 | 76 |
| Caulosphere | B125 | Naju | 51649 | 30030 | 64 |
| Caulosphere | B126 | Gurye | 48992 | 860 | 50 |
| Caulosphere | B127 | Gurye | 51303 | 10287 | 149 |
| Caulosphere | B128 | Gurye | 86422 | 1488 | 102 |
| Caulosphere | B129 | Gurye | 59257 | 5701 | 314 |
| Caulosphere | B130 | Gurye | 46983 | 258 | 27 |
| Caulosphere | B131 | Sangju | 41667 | 228 | 21 |
| Caulosphere | B132 | Sangju | 43720 | 613 | 46 |
| Caulosphere | B133 | Sangju | 47508 | 347 | 25 |
| Caulosphere | B134 | Sangju | 51521 | 1215 | 64 |
| Caulosphere | B135 | Sangju | 56074 | 669 | 52 |
| Caulosphere | B136 | Seosan | 53633 | 772 | 45 |
| Caulosphere | B137 | Seosan | 49048 | 299 | 22 |
| Caulosphere | B138 | Seosan | 51764 | 145 | 9 |
| Caulosphere | B139 | Seosan | 38196 | 1421 | 48 |
| Caulosphere | B140 | Seosan | 42598 | 538 | 32 |
| Caulosphere | B141 | Buyeo | 42074 | 1146 | 47 |
| Caulosphere | B142 | Buyeo | 65282 | 64565 | 20 |
| Caulosphere | B143 | Buyeo | 47943 | 5011 | 91 |
| Caulosphere | B144 | Buyeo | 63554 | 6184 | 78 |
| Carposhere | B121 | Naju | 54901 | 9282 | 104 |
| Carposhere | B122 | Naju | 45899 | 213 | 12 |
| Carposhere | B123 | Naju | 44265 | 210 | 15 |
| Carposhere | B124 | Naju | 59741 | 282 | 25 |
| Carposhere | B125 | Naju | 46440 | 338 | 23 |
| Carposhere | B126 | Gurye | 51310 | 2720 | 19 |
| Carposhere | B127 | Gurye | 56924 | 1209 | 17 |
| Carposhere | B128 | Gurye | 68044 | 848 | 21 |
| Carposhere | B129 | Gurye | 56100 | 1130 | 28 |
| Carposhere | B130 | Gurye | 56416 | 21757 | 23 |
| Carposhere | B131 | Sangju | 64896 | 2199 | 21 |
| Carposhere | B132 | Sangju | 44053 | 750 | 25 |
| Carposhere | B133 | Sangju | 51129 | 325 | 18 |
| Carposhere | B134 | Sangju | 45273 | 203 | 12 |
| Carposhere | B135 | Sangju | 46562 | 3825 | 14 |
| Carposhere | B136 | Seosan | 70117 | 599 | 19 |
| Carposhere | B137 | Seosan | 53503 | 1616 | 14 |
| Carposhere | B138 | Seosan | 45653 | 1087 | 7 |
| Carposhere | B139 | Seosan | 46798 | 287 | 25 |
| Carposhere | B140 | Seosan | 47326 | 132 | 9 |
| Carposhere | B141 | Buyeo | 43794 | 103 | 5 |
| Carposhere | B142 | Buyeo | 45227 | 174 | 13 |
| Carposhere | B143 | Buyeo | 50303 | 908 | 68 |
| Carposhere | B144 | Buyeo | 56582 | 391 | 27 |
| Anthosphere | B121 | Naju | 50061 | 3004 | 171 |
| Anthosphere | B122 | Naju | 54755 | 108 | 5 |
| Anthosphere | B123 | Naju | 45356 | 103 | 9 |
| Anthosphere | B124 | Naju | 44878 | 37264 | 21 |
| Anthosphere | B125 | Naju | 49926 | 817 | 38 |
| Anthosphere | B126 | Gurye | 46456 | 1856 | 18 |
| Anthosphere | B127 | Gurye | 62635 | 129 | 8 |
| Anthosphere | B128 | Gurye | 53886 | 201 | 19 |
| Anthosphere | B129 | Gurye | 51395 | 568 | 11 |
| Anthosphere | B130 | Gurye | 53127 | 461 | 37 |
| Anthosphere | B131 | Sangju | 45836 | 459 | 21 |
| Anthosphere | B132 | Sangju | 45979 | 1108 | 93 |
| Anthosphere | B133 | Sangju | 49175 | 503 | 24 |
| Anthosphere | B134 | Sangju | 50728 | 519 | 20 |
| Anthosphere | B135 | Sangju | 51408 | 1248 | 79 |
| Anthosphere | B136 | Seosan | 60308 | 193 | 19 |
| Anthosphere | B137 | Seosan | 52568 | 679 | 15 |
| Anthosphere | B138 | Seosan | 56579 | 109 | 7 |
| Anthosphere | B139 | Seosan | 42094 | 1301 | 21 |
| Anthosphere | B140 | Seosan | 51083 | 268 | 29 |
| Anthosphere | B141 | Buyeo | 54461 | 313 | 35 |
| Anthosphere | B142 | Buyeo | 49346 | 1719 | 101 |
| Anthosphere | B143 | Buyeo | 52686 | 6183 | 308 |
| Anthosphere | B144 | Buyeo | 67996 | 1199 | 83 |


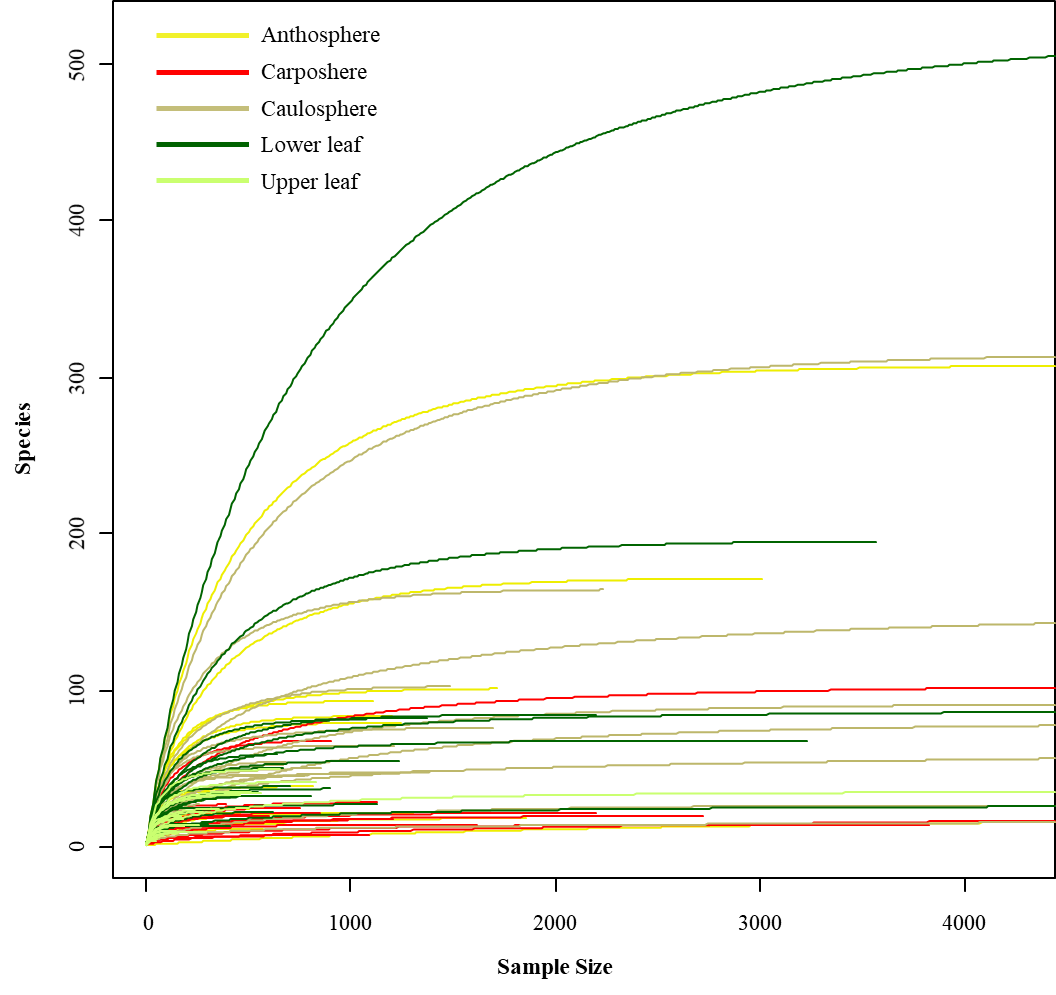


**Figure S1.** Rarefaction curves of phyllosphere samples in this study.

**
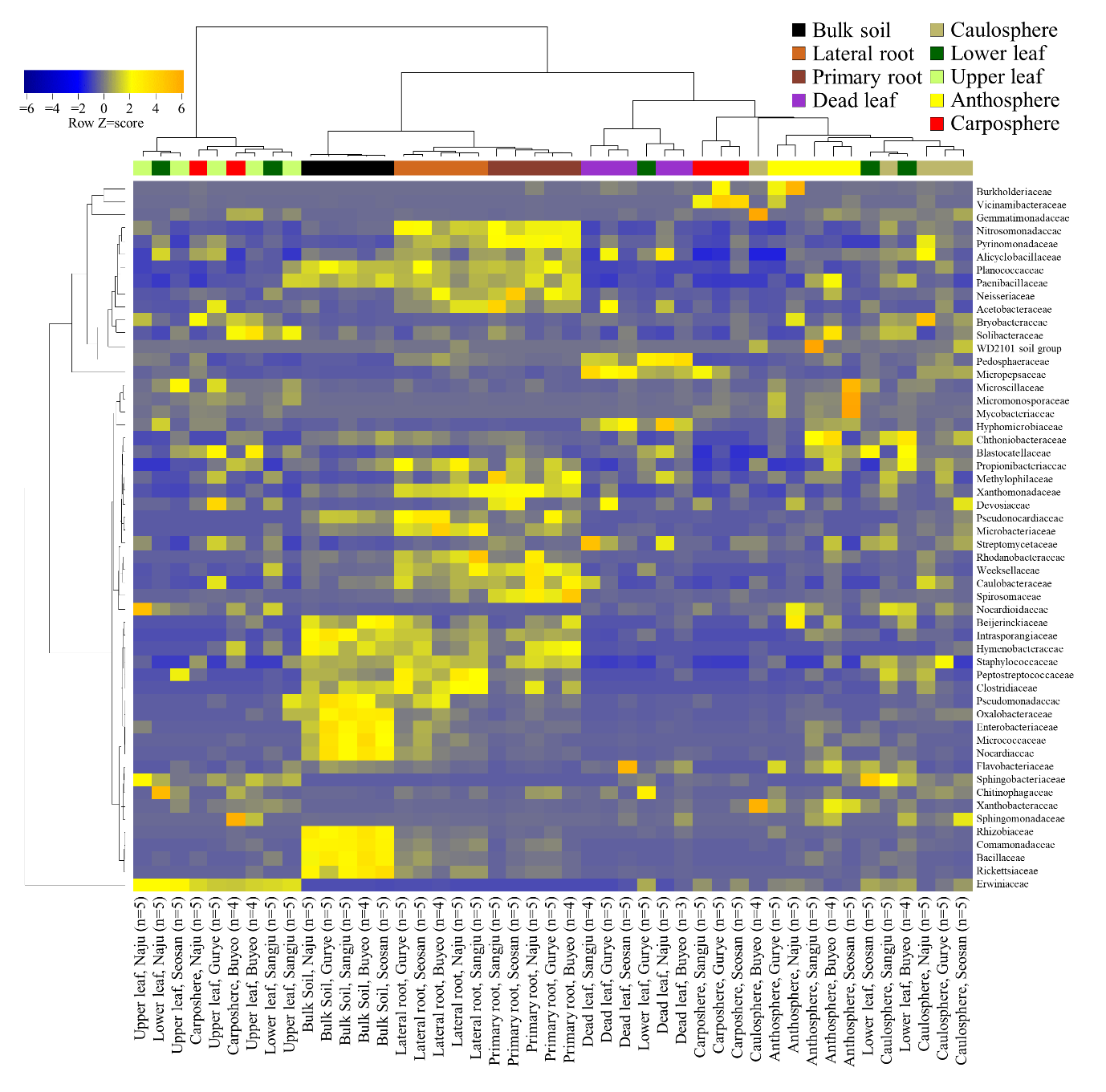
**

**Figure S2.** Heatmap of hierarchical clustering of major bacterial groups at the genus level. Heatmap color (blue to yellow) displays the row scaled relative abundance (Row Z-score) of each taxon across all samples.


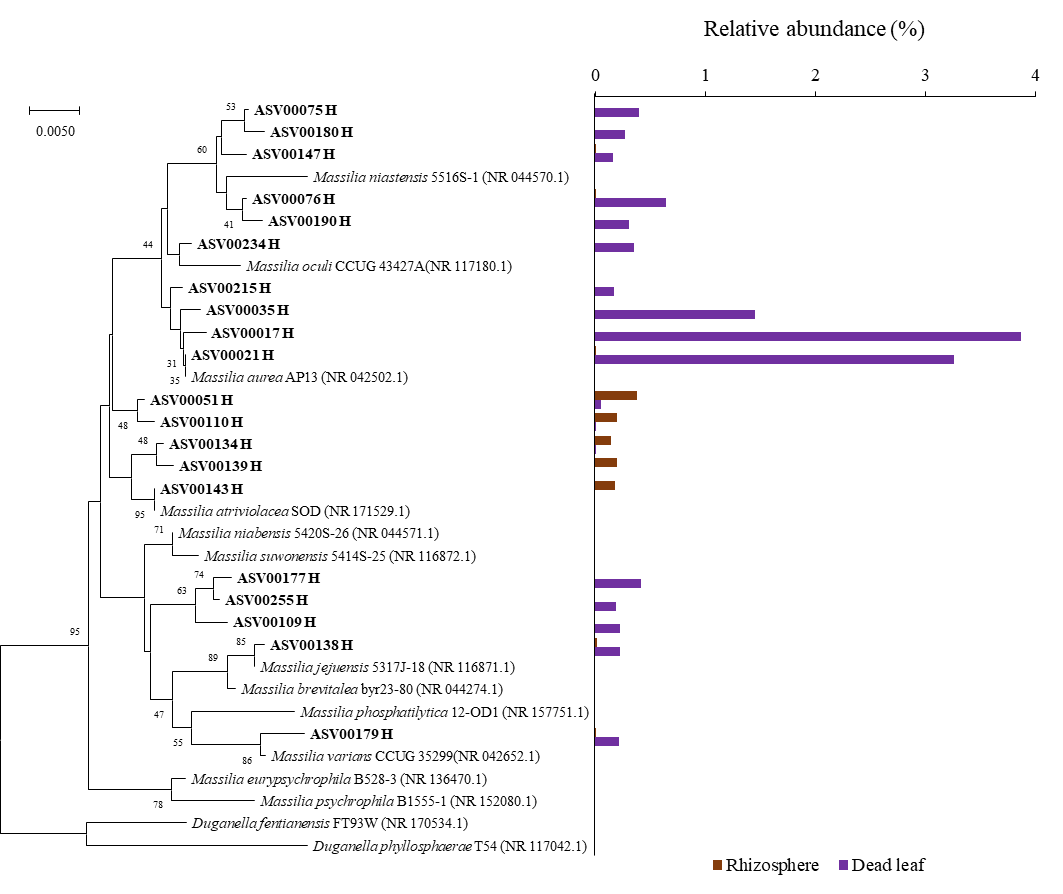


**Figure S3.** Phylogenic tree based on the neighbor-joining method and the relative abundance of *Massilia* ASVs in the rhizosphere and dead leaf samples.
